# Estimating Left Atrial Pressure Using Diastolic Cutoff Values After Transcatheter Mitral Valve Edge-to-Edge Repair

**DOI:** 10.3390/jcm14124308

**Published:** 2025-06-17

**Authors:** Yoav Niv Granot, Giulia Passaniti, Samin Sharma, Annapoorna Kini, Daniel Karlsberg, Sahil Khera, Gilbert H. L. Tang, Stamatios Lerakis, Lucy M. Safi

**Affiliations:** 1 Mount Sinai Fuster Heart Hospital, Icahn School of Medicine at Mount Sinai, New York, NY 10029, USA; 2 Leon H. Charney Division of Cardiology, Department of Medicine, NYU Langone Health, New York, NY 10016, USA; 3 Department of Cardiovascular Surgery, Mount Sinai Health System, New York, NY 10029, USA

**Keywords:** transcatheter mitral valve edge-to-edge repair, left atrial pressure, diastolic function, mitral regurgitation

## Abstract

**Background:** Transcatheter mitral valve edge-to-edge repair (M-TEER) reduces mitral regurgitation (MR) severity and improves symptoms, but early post-procedural assessment of left atrial pressure (LAP) remains challenging. **Objectives**: Investigating the impact of M-TEER on diastolic parameters and derived cutoff values to estimate post-procedural LAP. **Methods**: This retrospective study (n = 240) analyzed the effects of M-TEER on diastolic parameters. Cutoff values for predicting normal LAP were identified using classification tree analysis and validated with current methods for assessing LAP. **Results**: M-TEER increased E/e′ ratio in both normal and abnormal left ventricular ejection fraction (LVEF) groups. In normal LVEF, E wave velocity ≤ 85 cm/s at 30 days correlated with normal LAP (98% specificity, 90% positive predictive value). In abnormal LVEF, E/e′ ≤ 14 or E wave velocity ≤ 95 cm/s correlated with normal LAP (98%/90% specificity, 91%/83% positive predictive value). The validation of the proposed cutoff values with existing non-invasive methods showed 96% accuracy for normal LAP (24/25) and 90% for elevated LAP (27/30). **Conclusions**: M-TEER significantly alters diastolic parameters. Derived cutoff values based on easily obtainable diastolic measures show promise in estimating post-procedural LAP, but need further validation for clinical use.

## 1. Introduction

Echocardiographic assessment of left ventricular (LV) diastolic function plays a crucial role in the comprehensive evaluation of patients, particularly those with suspected heart failure or symptoms suggestive of heart disease [1,2]. LV diastolic dysfunction, characterized by impaired relaxation and increased stiffness of the heart muscle, leads to elevated filling pressures that ultimately contribute to congestive symptoms.

While current American Society of Echocardiography (ASE) and European Association of Cardiovascular Imaging (EACVI) guidelines for determining left ventricular (LV) filling pressures and diastolic function are generally applicable, they may not accurately reflect these parameters in patients who have undergone mitral valve intervention [1,2]. Several key indicators included in the guidelines, such as maximal early diastolic flow (E wave), mitral annulus velocities (e′) and the E/e′ ratio, can be significantly altered by surgical interventions that modify the mitral valve annulus. For example, the placement of a surgical ring or mitral prosthesis can inherently lower e′ values, even if intrinsic LV relaxation remains normal. Therefore, it is crucial to consider the impact of mitral valve intervention on echocardiographic parameters when assessing LV filling pressures and diastolic function.

Transcatheter mitral valve edge-to-edge repair (M-TEER) offers significant benefits for both primary and secondary mitral regurgitation (MR) patients, demonstrably reducing symptoms, MR severity, left ventricular dimensions, surgical needs and hospitalizations [3,4]. Notably, post-procedure left atrial pressure (LAP) emerges as a key predictor of clinical outcomes and symptomatic improvement [5,6,7].

The newly published ASE guideline for evaluation of valvular abnormalities after M-TEER only acknowledges pulmonary vein flow and pulmonary artery systolic pressure as markers of elevated left sided filling pressure [8]. Other promising parameters are emerging, with studies suggesting that peak flow through residual atrial septal defects [9,10] may offer valuable information regarding LAP.

The primary aim of this assessment was to identify non-invasive markers that reliably reflect left atrial pressure (a key determinant of congestion) by determining the predictive value of classic diastolic parameters (E/e′, E, LAVI) for estimating left atrial pressure after M-TEER, and comparing their accuracy with existing methods (pulmonary vein flow and peak ASD flow).

## 2. Materials and Methods

This retrospective analysis, conducted at a single, high-volume tertiary center [11], investigated patients who underwent M-TEER with the MitraClip device (Abbott Vascular, Santa Clara, CA, USA) between January 2017 and December 2022. Patients with prior mitral valve interventions (surgical ring, n = 3; prior M-TEER, n = 9) or missing left atrial pressure (LAP) measurements (n = 2) were excluded.

The study was reviewed and approved by the Institutional Review Board with a waiver of informed consent.

### 2.1. M-TEER Procedure

M-TEER was performed with the MitraClip device in the cardiac catheterization laboratory under general endotracheal anesthesia for all patients. Intraprocedural transesophageal echocardiography (TEE) guidance was performed by advanced structural heart imagers using the Phillip’s EPIQ CVx system (Phillips, Andover, MA, USA). Transseptal puncture was achieved using standard techniques with TEE guidance, aiming for a posterior and mid-to-superior location on the interatrial septum. Adequate septal distance from the puncture site to the mitral annular plane was confirmed prior to puncture. After transseptal puncture, baseline LAP and V waves were documented.

The steerable MitraClip guide sheath was advanced into the left atrium (LA) under fluoroscopic and TEE guidance. The MitraClip delivery system was carefully navigated through the LA under TEE guidance to the optimal position above the mitral valve. The clip was oriented perpendicular to leaflet coaptation plane at the level of the largest jet using 3D TEE imaging. The clip was then advanced into the left ventricle and pulled back to grasp the mitral valve leaflets using TEE guidance. Evaluation of MR reduction, mitral inflow gradient, and tissue bridge on 3D imaging and pulmonary venous flow pattern were evaluated prior to clip deployment. If moderate or significant residual MR remained after the first device deployment, additional MitraClip placement was considered.

### 2.2. LA Pressure Measurement

Direct left atrial mean pressure and V wave were measured after transseptal puncture (baseline measurement) and at the end of the procedure following MitraClip deployment.

### 2.3. Echocardiography

As part of routine clinical care, Patients were planned for a comprehensive pre-procedure (Pre), and 30-day post-procedure (POD30) transthoracic echocardiograms to evaluate procedural success following contemporary guidelines [1,2,8]. MR severity was determined by an integrative approach combining semi-quantitative and quantitative parameters: assessment of vena contracta width, MR jet area, effective orifice area (EROA) and regurgitant volume by the PISA method.

Pulsed-wave Doppler echocardiography was performed in the apical four-chamber view to assess LV filling by measuring mitral inflow velocities. A 1 mm to 3 mm sample volume was placed between the mitral leaflet tips at end-expiration and during diastole after optimizing spectral gain, wall filter settings, and setting sweep speeds of 100 mm/s. Recordings were averaged over three consecutive cardiac cycles in patients with sinus rhythm and over five cycles in patients with atrial fibrillation (AF) for improved accuracy. Measurements of mitral inflow included E wave, A wave and E/A ratio. Early diastolic mitral annular velocities (e′) were measured in the apical 4-chamber view using tissue Doppler from the septal and lateral annuli. Mitral E/e′ ratio was calculated from the average of at least 3 cardiac cycles.

Left atrium volume was calculated using biplane method of disks by tracing the endocardial borders at end-systole in both the apical four-and two-chamber views. The left atrial volume index (LAVI) was calculated by adjusting the volume for the patient’s body surface area (BSA).

Left ventricular end systolic and diastolic diameter (LVESD, LVEDD) were measured in the parasternal view at the level of the mitral valve leaflet tips. The left ventricular ejection fraction (LVEF) was calculated using Simpson’s summation of disks method, except for a minority of cases (n = 53) where visual estimation was employed. Abnormal LVEF was defined as <52% for male and <54% for female [12].

Right ventricular (RV) size and function were assessed in multiple views by a specialized imaging cardiologist who provided an integrative qualitative and quantitative grading of RV function into this categories: Normal function, Mild, Mild to Moderate, Moderate or greater than moderate RV dysfunction.

Postprocedural peak velocity of the pulmonary vein flow spectral Doppler waveform was recorded during systole and diastole. Pulmonary venous flow morphology for each patient was categorized as systolic dominant, systolic blunting, or systolic reversal. If more than one pulmonary vein was assessed, the vein exhibiting the most abnormal morphology was selected. Two categories were then created: normal pattern morphology (defined as systolic dominant) and abnormal morphology (defined as systolic blunting or systolic reversal morphology).

### 2.4. Statistical Analysis

Categorical variables reported as numbers and percentages, and continuous variables reported as means and standard deviations or medians and interquartile ranges (IQRs), as appropriate. Continuous variables tested for normal distribution using histograms, Q-Q Plots and normality tests (Shapiro–Wilk). Continuous variables compared between groups using independent samples *t*-test, ANOVA or Mann–Whitney test and categorical variables compared using Chi-square test or Fisher’s exact test.

For each LVEF group (normal and abnormal), we compared changes in various echocardiographic measurements over time using either paired *t*-tests (for normally distributed data) or paired-samples Wilcoxon signed-rank tests (for non-normally distributed data). Patients with residual MR that was graded as moderate or higher were excluded from this comparison.

To identify diastolic parameters associated with normal post-procedure left atrial pressure (LAP, defined as <15 mmHg), we performed a stratified analysis for each parameter (E/e′, max E wave velocity, DT, TR peak velocity). This analysis was performed separately for normal and abnormal LVEF groups. We used either a chi-squared automatic interaction detection model (CHAID) or a classification and regression tree (CART) model with specific criteria: maximum tree depth of 3 levels, minimum of 30 cases in parent nodes, and minimum of 10 cases in child nodes. The first node divided the cohort into normal and abnormal left ventricular ejection fraction (LVEF) groups.

Both models automatically selected the best predictors for splitting the data into child nodes, with significance level provided for each branch. To assess the models’ performance, we calculated the area under the receiver operating characteristic curve (AUC) and evaluated sensitivity, specificity, positive predictive value (PPV), negative predictive value (NPV), and accuracy for each cutoff value in predicting normal post-procedure LAP.

To validate our cutoff values for left atrial pressure (LAP) assessment after a procedure, we compared them to two non-invasive established methods:

− Pulmonary vein flow pattern: Blunted systolic flow pattern suggests elevated LAP.
− Max flow velocity through residual ASD combined with estimated right atrial pressure: An estimated pressure less than 15 mmHg is considered normal LAP.

In cases of disagreement between these two methods, the pressure derived from the residual ASD was preferred.

All statistical tests were two-sided, and a *p*-value of <0.05 was considered statistically significant. SPSS software was used for all statistical analysis (IBM SPSS statistics, version 29).

## 3. Results

### 3.1. Study Population

A total of 240 patients were included in the study, divided into two groups: 122 with abnormal LVEF and 118 with normal LVEF (Table 1). The median age across both groups was 82.5 years with 49% female patients and a median STS score of 6.74%. Compared to those with normal LVEF, patients with reduced LVEF were slightly younger (median age 80.9 vs. 84.6 years, *p* = 0.004) and less likely to be female (39% vs. 58%, *p* = 0.003). They also had a higher STS score (7.44% vs. 5.87%, *p* = 0.006), higher baseline creatinine (1.4 mg/dL vs. 1.1 mg/dL, *p* < 0.001), and were more likely to have comorbidities such as diabetes mellitus, coronary artery disease, and coronary intervention. Patients with normal EF were more likely to be classified as frail.

Pre-procedural echocardiographic measurements for the entire cohort and stratified by LVEF group can be found in Table 2. Notably, the estimated MR EROA by PISA method was similar between groups (0.4 cm^2^ overall, 95% IQR 0.3–0.5 cm^2^, *p* = 0.062). The regurgitant volume was slightly higher in the normal LVEF group (67.5 mL vs. 61 mL, *p* = 0.013).

Patients with abnormal LVEF displayed enlarged left ventricles and a higher prevalence of right ventricular dysfunction compared to those with normal LVEF. However, no significant differences were observed in left-sided valvular abnormalities between the groups.

### 3.2. Procedural Characteristics

Both groups experienced significant MR reduction (Table 3).

Central clip placement (A2–P2 location) was the predominant approach, with a slightly higher frequency in the abnormal LVEF group (97% vs. 89%, *p* = 0.019). Among the patients, 54% (n = 130) achieved normalized LAP at the end of the procedure, with similar proportions observed in both LVEF groups.

### 3.3. Effect of M-TEER on Diastolic Parameters Between Baseline and POD30

Maximal E wave velocity showed a subtle upward trend, statistical significance for patients with abnormal LVEF (111 vs. 100 cm/s, *p* = 0.044). Interestingly, lateral e′ decreased in both groups, while septal e′ reduction was seen only in patients with normal LVEF. This resulted in a higher E/e′ ratio for both groups (Table 4).

Both tricuspid regurgitation peak velocity and LAVI remained similar.

### 3.4. Cutoff Predictive for Normalization of Left Atrium Pressure

Table A1 shows a comparison of pre-procedural echocardiographic characteristics in each LV function group based on post-procedural normalization of left atrial pressure. No significant differences in baseline diastolic parameters were observed between LVEF function groups.

A classification tree analysis identified potential cutoff values for each LV function group associated with normalization of left atrial pressure (invasive measurement at the end of the procedure). For patients with normal LVEF, the analysis suggested cutoff values for maximum E wave velocity of 85 cm/s and 163 cm/s). For patients with abnormal LVEF, the analysis suggested cutoff values for maximum E wave velocity of 95 cm/s and 158 cm/s and E/e′ ratio of 14 and 34.

Table 5 shows the discriminative ability of each cutoff value using the AUC. For patients with normal baseline LVEF, maximum E wave velocity cutoffs had the best discriminative power (AUC 0.723, IQR 0.584–0.862, *p* = 0.002). For patients with abnormal baseline LVEF, E/e′ ratio cutoffs achieved the highest AUC (0.861, IQR 0.732–0.991, *p* < 0.001).

Detailed sensitivity, specificity, PPV, NPV, and accuracy for each cutoff value with regard to normal invasive LAP at the end of the procedure are presented in Table 6.

An E/e′ ratio ≤ 14 demonstrated a high specificity (98%) and PPV of 91%, indicating a high likelihood of LAP normalization if this criterion was met. Alternatively, a maximum E wave velocity ≤ 95 cm/s exhibited high specificity (90%) and PPV of 83%, suggesting a similar interpretation. Conversely, a maximum E wave velocity < 158 cm/s and an E/e′ < 34 achieved high sensitivity (96% and 94%, respectively) and NPV of 86% and 85%, respectively. These values suggest a low probability of LAP normalization if these criteria are not met.

For patients with normal baseline LVEF, a maximum E wave velocity ≤ 85 cm/s demonstrated high specificity (98%) and PPV of 90%, indicating a high likelihood of LAP normalization if this criterion is met. Conversely, a maximum E wave velocity < 163 cm/s achieved high sensitivity (86%) and NPV of 69%, suggesting a low probability of LAP normalization if these criteria are not met.

### 3.5. Validation of the Suggested Cutoff Values

To validate the suggested cutoff values, we analyzed 99 patients with less than moderate residual MR at 30-day follow-up. These patients had both diastolic data and either pulmonary vein flow pattern or peak velocity through the residual ASD to assess LAP non-invasively (Table 7). The cutoff values correctly identified 96% of patients with normal LAP (24/25) and 90% of patients with elevated LAP (27/30), based on these established methods.

**Figure 1:**
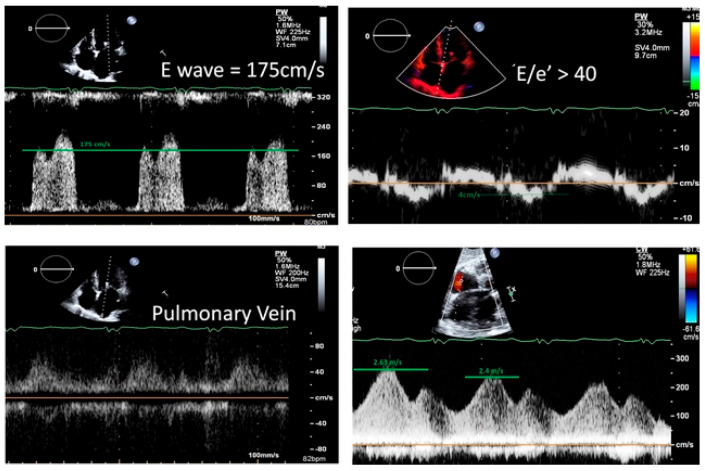
Representative case of patient with abnormal LVEF (LVEF = 45%) post M-TEER (at A2–P2) with good results (mild to moderate residual jet). While PV flow pattern may suggest normal LAP, peak ASD flow and our suggested diastolic parameters (E/e′ > 40) more compatible with elevated LAP.

Representative case of assessment of LAP post procedure based on current methods and suggested diastolic cutoff values is shown in Figure 1.

## 4. Discussion

This study investigates the early post-procedural impact of M-TEER on classic diastolic function parameters. Although no statistically significant change in E wave velocity was observed, we identified a trend towards higher E wave alongside a statistically significant decrease in e′ velocity compared to baseline TTE. This resulted in a significantly higher E/e′ ratio post-procedure. Notably, LAVI remained unchanged in the short term.

Based on our analysis, the following preliminary cutoff values may be used to assist in estimating post-procedural LAP after M-TEER.

For patients with normal baseline LVEF:

- Elevated LAP: Maximum E wave velocity > 163 cm/s
- Normal LAP: Maximum E wave velocity ≤ 85 cm/s

For patients with abnormal baseline LVEF:

- Elevated LAP: Maximum E wave velocity > 158 cm/s OR E/e′ ratio > 34
- Normal LAP: Maximum E wave velocity ≤ 95 cm/s OR E/e′ ratio ≤ 14

The proposed 30 days cutoff value for LAP demonstrated high accuracy compared with current non-invasive methods (pulmonary vein flow pattern or peak flow through residual ASD), achieving 96% accuracy for normal LAP and 90% accuracy for elevated LAP.

LAP serves as a critical indicator of the combined impact of various factors affecting the heart after M-TEER and LAP ultimately influences a patient’s clinical outcomes and symptoms post-procedure [13,14]. Although invasive measurements of LAP are routinely performed during M-TEER procedure, there remains a critical lack of reliable, non-invasive methods for monitoring LAP using echocardiography after the procedure. This is due to limitations inherent in using mitral annulus and inflow velocities after valve manipulation [15].

Current guidelines suggest pulmonary vein flow pattern as a surrogate marker for post M-TEER left atrial pressure normalization, despite limitations in its application [8]. While Corrigan et al. demonstrated a correlation between improved pulmonary vein flow and reduced heart failure hospitalization, this method has two key drawbacks: (1) only 27% of patients exhibit normal flow post-procedure [14] and (2) evaluating pulmonary vein flow with TTE can be challenging [16]. Alternative approaches for estimating LAP exist, such as measuring flow through residual atrial septal defects [9,10] face limitations in terms of widespread applicability and feasibility.

Our study findings concerning the impact of M-TEER on diastolic parameters align with previous reports [17,18,19]. We observed increases in maximum E wave velocity alongside reductions in mitral annulus velocities, leading to elevated E/e′ ratios. The influence of M-TEER on LAVI is more nuanced, with some studies finding no significant changes [20] and others reporting volume reductions only in long-term follow-up [17,21]. In line with these findings, our study observed no significant change in LAVI within the short-term (up to 30 days post-procedure). Importantly, this study uniquely investigates the identification of diastolic cutoff values for predicting post-M-TEER LAP in both normal and abnormal LVEF patients. To our knowledge, this represents the first attempt to achieve this.

### Limitations

Our study has several limitations that should be considered when interpreting the findings. This is a single-center, retrospective study that limits the generalizability of our findings to other populations and settings. Conducting a multicenter study would increase the generalizability of our results. One M-TEER device system was used in this study and its applicability to other available M-TEER devices is unknown. Although our study was completed at a busy tertiary care center, our sample size remains relatively small which restricted our ability to perform more advanced analyses, such as dividing the cohort into separate learning and validation groups. This could potentially affect the reliability and robustness of our findings. Furthermore, due to the retrospective nature of this analysis, a number of patients had missing diastolic data during the follow-up period.

Utilizing intraprocedural left atrial pressure (LAP) measurements for defining normalization has limitations. Interprocedurally, general anesthesia can influence hemodynamics and potentially affect LAP values. Post-procedurally, LAP can fluctuate significantly during the follow-up period. While our suggested cutoff values were derived from invasive measurements obtained at the end of the procedure, their correlation with alternative methods at 30 days follow up (PV flow pattern, peak ASD flow) strengthened our findings. It should be noted that invasive LAP monitoring is not routinely performed after M-TEER procedures, Therefore, indirect echocardiographic parameters were used as alternatives.

## 5. Conclusions

Currently, the non-invasive assessment of left atrial pressure (LAP) has limited options and may not be feasible in many patients. This study demonstrates a significant impact of M-TEER on diastolic parameters, particularly early mitral inflow, mitral annulus velocities, and the resulting E/e′ ratio. We identified potentially useful cutoff values derived from these easily obtainable diastolic measures that may assist in predicting post-procedural LAP. Building upon these important achievements, future studies are now essential to rigorously validate these promising findings and, most importantly, to confirm their broader clinical utility and applicability in patient management.

## Figures and Tables

**Table 1:** Baseline clinical characteristics in the entire cohort and according to LVEF.

	All (n = 240)	Abnormal LVEF (n = 122)	Normal LVEF (n = 118)	*p* Value
Age, years *	82.5 (74.4–87.4)	80.9 (72.3–85.8)	84.6 (75.4–89.2)	0.004
Female, %	117 (49)	48 (39)	69 (58)	0.003
BSA, m^2^	1.8 ± 0.26	1.8 ± 0.25	1.7 ± 0.26	0.003
STS–Mitral Valve Repair, % *	6.74% (3.64–10.5)	7.44% (3.98–11.65)	5.87% (3.07–9)	0.006
Baseline Creatinine, mg/dL *	1.3 (1–1.8)	1.4 (1.1–2)	1.1 (0.9–1.5)	<0.001
Frailty, %	183 (76)	84 (69)	99 (84)	0.006
Scoliosis, %	13 (5)	1 (1)	12 (10)	0.001
CAD, %	92 (38)	62 (51)	30 (25)	<0.001
Diabetes mellitus, %	53 (22)	34 (28)	19 (16)	0.028
CVA/TIA, %	33 (14)	17 (14)	16 (14)	0.933
PVD, %	23 (10)	14 (11)	9 (8)	0.311
COPD, %	61 (26)	29 (24)	32 (27)	0.268
Immunocompromised, %	16 (7)	8 (7)	8 (7)	0.945
Atrial fibrillation, %	116 (48)	64 (52)	52 (44)	0.193
Dialysis, %	26 (11)	21 (17)	5 (4)	0.001
Liver disease, %	7 (3)	2 (2)	5 (4)	0.232
Porcelain Aorta, %	4 (2)	3 (2)	1 (1)	0.33
Prior PCI, %	57 (24)	45 (37)	12 (10)	<0.001
Cardiac leads, %	58 (24)	44 (36)	14 (12)	<0.001

* Median and interquartile range. All other values represent the number of patients and percentages.

**Table 2:** Pre-procedural echocardiographic characteristics in the entire cohort and according baseline LVEF.

		All (n = 240)	Abnormal LVEF (n = 122)	Normal LVEF (n = 118)	*p* Value
LVEF, % *		52.5 (35–61)	35 (25–45)	61.5 (58–65)	<0.001
LVEDD, cm *		5.2 (4.6–5.9)	5.6 (5.2–6.4)	4.9 (4.4–5.3)	<0.001
LVESD, cm *		3.7 (3.1–4.5)	4.5 (3.9–5.5)	3.2 (2.9–3.7)	<0.001
LV systolic volume, mL *		64.5 (40–109.9)	108 (68–157)	41 (32–60)	<0.001
LV diastolic volume, mL *		138 (96–196)	176.2 (124–231)	113.5 (84–143)	<0.001
Mitral regurgitation EROA, cm^2^ *		0.4 (0.3–0.5)	0.4 (0.3–0.5)	0.4 (0.3–0.5)	0.062
Mitral regurgitation Regurgitant volume, mL *		64 (52–78)	61 (50–75)	67.5 (57–81)	0.013
Mitral regurgitation etiology	Primary	96 (40)	18 (15)	78 (66)	<0.001
	Secondary	144 (60)	104 (85)	40 (34)	<0.001
Tricuspid regurgitation, %	None/Trace	23 (10)	13 (11)	10 (9)	0.38
	≤Moderate	164 (68)	86 (70)	78 (65)	
	Moderate to Severe	27 (11)	12 (10)	15 (13)	
	Severe	26 (11)	11 (9)	15 (13)	
Aortic regurgitation, %	None/Trace	122 (51)	67 (55)	55 (47)	0.43
	≤Moderate	116 (48)	54 (44)	62 (53)	
	>Moderate	2 (1)	1 (1)	1 (1)	
Right ventricle size, %	Normal	146 (61)	69 (56)	77 (65)	0.57
	≤Moderate	82 (34)	46 (38)	36 (31)	
	>Moderate	12 (5)	7 (6)	5 (4)	
Right ventricle function, %	Normal	137 (57)	53 (43)	82 (69)	<0.001
	≤Moderate	90 (38)	60 (49)	32 (27)	
	>Moderate	13 (5)	9 (7)	4 (3)	

* Median and interquartile range. All other values represent the number of patients and percentages.

**Table 3:** Procedural characteristics in the entire cohort and according baseline LVEF.

		All (n = 240)	Abnormal LVEF (n = 122)	Normal LVEF (n = 118)	*p* Value
Left atrium pressure (baseline), mmHg *		20 (15–25)	20 (15–25)	20 (15–25)	0.435
Left atrium V wave (baseline), mmHg *		35 (25–46)	35 (25–45)	40 (30–47)	0.122
Clip Location	A1–P1	13 (5)	2 (2)	11 (9)	0.009
	A2–P2	223 (93)	118 (97)	105 (89)	0.019
	A3–P3	18 (8)	5 (4)	13 (11)	0.042
Clip generation (G4)		70 (29)	32 (26)	38 (32)	NS
Left atrium pressure (procedure end), mmHg *		13 (10–18)	14 (10–18)	13 (10–16)	0.95
Left atrium pressure change, mmHg *		6 (4–10)	6 (5–10)	7 (4–10)	0.465
Left atrium V wave (procedure end), mmHg *		20 (15–25)	14 (10–18)	13 (10–16)	0.725
Left atrium V wave pressure change, mmHg *		15 (10–20)	13 (9–20)	15 (10–25)	0.124
Normal Left atrium pressure post procedure (<15 mmHg)		130 (54)	62 (51)	68 (58)	0.29

* Median and interquartile range. All other values represent the number of patients and percentages.

**Table 4:** Comparison of Pre-procedural and Post Procedure Diastolic Parameters within Each LVEF group.

	Abnormal LVEF			Normal LVEF		
	**Pre**	**30 Day** **Follow Up**	***p***	**Pre**	**30 Day** **Follow Up**	***p***
Mitral regurgitation grade	4 (3.5–4)	2 (2–2.5)	<0.001	4 (3.5–4)	2 (2–2.5)	<0.001
Max E wave, cm/s *	100 (83–113)	111 (77.6–142)	0.044	107 (89.7–120.8)	125 (91–152)	0.08
Lateral e′, cm/s *	7 (5–10)	5.6 (3.9–7.1)	0.001	8 (6.2–9.5)	6 (5–7.3)	<0.001
septal e′, cm/s *	5 (3.9–6)	4.2 (3–5.2)	0.061	5.9 (4.4–6.6)	4.2 (3.4–5.1)	0.001
E/e′ *	16.8 (10.7–20.5)	24.3 (18–31.4)	0.001	15.1 (12.2–20.3)	22.9 (17–29.3)	<0.001
Tricuspid regurgitation peak velocity, cm/s *	3.1 (2.6–3.3)	3 (2.8–3.4)	0.853	3.2 (2.8–3.5)	3 (2.7–3.3)	0.567
Left atrium volume index, mL/m^2^ *	66.4 (46.6–83.1)	61.4 (44.7–86.6)	0.379	55.1 (44–75.9)	64.9 (40.1–72)	0.586

* Median and interquartile range. All other values represent the number of patients and percentages.

**Table 5:** Distribution of diastolic echocardiographic measurements according to normalization of left atrial pressure and the accuracy of discriminative measurements (area under the curve).

	Cut-Off	Normal LAP *	High LAP *	ROC ^†^	*p*
Normal LVEF					
E max, cm/s	≤85	9 (17)	1 (2.4)	0.723 (0.584–0.862)	0.002
	85–163	36 (68)	22 (54)		
	>163	8 (15)	18 (44)		
Abnormal LVEF					
E Max, cm/s	≤95	19 (37)	4 (10)	0.742 (0.57–0.914)	0.006
	95–158	31 (60)	26 (62)		
	>158	2 (4)	12 (29)		
E/e′ ratio	≤14	10 (20)	1 (2)	0.861 (0.732–0.991)	<0.001
	14–34	37 (74)	23 (56)		
	>34	3 (6)	17 (42)		

* Values represent the number of patients and percentages. ^†^ 95% confidence interval.

**Table 6:** The sensitivity, specificity, PPV, NPV, and accuracy of each cutoff value with regard to normal invasive LAP at the end of the procedure.

	Sensitivity	Specificity	PPV	NPV	Accuracy
Normal LVEF					
E max ≤ 85 cm/s	20	98	90	52	52
E max < 163 cm/s	85	44	66	69	67
Abnormal LVEF					
E max ≤ 95 cm/s	37	90	83	54	61
E max < 158 cm/s	96	29	63	86	74
E/e′ ≤ 14	20	98	91	50	55
E/e′ < 34	94	41	66	85	70

**Table 7:** Relationship between suggested diastolic cutoff and left atrial pressure assessed by PV flow pattern or peak ASD flow.

		LAP Based on PV/ASD Flow	
LAP based on suggested cutoff values	Total	Normal *	Elevated *
Normal ^†^	25	24 (96)	1 (4)
Indeterminate	44	22 (50)	22 (50)
Elevated ^‡^	30	3 (10)	27 (90)

* Values represent the number of patients and percentages. ^†^ Maximum E wave velocity ≤ 85 cm/s (for patients with normal baseline LVEF), Maximum E wave velocity ≤ 95 cm/s or E/e′ ratio ≤ 14 (for patients with abnormal baseline LVEF). ^‡^ Maximum E wave velocity > 163 cm/s (for patients with normal baseline LVEF). Maximum E wave velocity > 158 cm/s or E/e′ ratio > 34 (for patients with abnormal baseline LVEF).

## Data Availability

Data generated or analyzed during the study are available from the corresponding author by request.

## Abbreviations

The following abbreviations are used in this manuscript:

LVEF	Left ventricle ejection function
BSA	Body surface index
STS	Society of thoracic surgeons
CAD	coronary artery disease
CVA	cerebrovascular accident
TIA	Transient ischemic attack
PVD	peripheral vascular disease
COPD	chronic obstructive pulmonary disease
PCI	Percutaneous coronary intervention
LVEDD	Left ventricle end diastolic diameter
LVESD	Left ventricle end systolic diameter
EROA	Effective regurgitant orifice area
LAP	Left atrial pressure
PPV	positive predictive value
NPV	negative predictive value
PV	Pulmonary vein
ASD	Atrial septal defect
M-TEER	Transcatheter mitral valve edge-to-edge repair
MR	Mitral regurgitation
LV	Left ventricle
TEE	Transesophageal echocardiography
LA	Left atrium
LAVI	Left atrial volume index
RV	Right ventricle
IQR	Interquartile range

## Appendix A

**Table A1:** Pre-procedural echocardiographic characteristics in the entire cohort and according to baseline LVEF and normalization of left atrium pressure at end of procedure.

	Abnormal LVEF			Normal LVEF		
	**Normal LAP**	**High LAP**	***p***	**Normal LAP**	**High LAP**	***p***
LVEF, %	35 (30–45)	35 (25–43.8)	0.263	65 (60–66.8)	60 (55.8–65)	0.037
LVEDD, cm	5.5 (5–6.2)	5.7 (5.4–6.5)	0.031	4.7 (4.4–5.3)	5 (4.5–5.5)	0.075
LVESD, cm	4.4 (3.9–5.4)	4.5 (3.9–5.6)	0.430	3.2 (2.6–3.7)	3.3 (3–3.7)	0.271
LV diastolic volume, mL	165 (117–201.4)	192 (144.5–261)	0.044	113.5 (82.1–145.3)	114.5 (87.8–142.9)	0.880
LV systolic volume, mL	99 (61–133)	123.5 (85.1–188.5)	0.021	41 (29.3–56.3)	44 (33–64)	0.440
Mitral regurgitation EROA, cm^2^	0.4 (0.3–0.5)	0.4 (0.3–0.5)	0.470	0.4 (0.3–0.6)	0.4 (0.3–0.5)	0.059
Mitral regurgitation regurgitant volume, mL	61.5 (52–74)	60 (47–79)	0.454	70 (61–86)	62 (52–76.5)	0.028
Left atrium volume index, mL/m^2^	63.7 (45.6–81.8)	64.3 (48.4–93.9)	0.620	59.3 (50.2–86.6)	61.5 (44.2–85.4)	0.589
Max E wave, cm/s	104.5 (88.4–122.5)	110 (95–122.3)	0.287	119 (96.8–135.5)	117 (102–160)	0.410
Max A wave, cm/s	64.8 (53.7–91.3)	69.8 (50.3–98.2)	0.925	80 (62–101.5)	73.3 (51.5–95.8)	0.378
E/A ratio	1.3 (1.1–2.1)	1.7 (1.3–2)	0.195	1.5 (1.1–1.8)	1.6 (0.9–2.4)	0.667
Lateral e′, cm/s	8 (5.9–11)	7.1 (6–9)	0.314	8.3 (7–11)	8 (6.9–9.6)	0.310
Septal e′, cm/s	5.1 (4–6.7)	5 (4–5.9)	0.562	6 (5.2–7.3)	5.8 (4.4–7.4)	0.534
E/e′	15.4 (11.9–20.9)	17.9 (13.6–22.5)	0.184	15.1 (11.9–20.6)	16.8 (13.5–20.7)	0.302
Tricuspid regurgitation peak velocity, cm/s	3.1 (2.7–3.3)	3.3 (2.9–3.5)	0.188	3.3 (3–3.9)	3.2 (2.8–3.5)	0.215

Median and interquartile range.
